# Diurnal patterns in Twitter sentiment in Italy and United Kingdom are correlated

**DOI:** 10.3389/fpsyg.2023.1276285

**Published:** 2024-01-19

**Authors:** Sheng Wang, Stafford Lightman, Nello Cristianini

**Affiliations:** ^1^School of Computer Science, University of Bristol, Bristol, United Kingdom; ^2^Henry Wellcome Laboratories for Integrative Neuroscience and Endocrinology, University of Bristol, Bristol, United Kingdom; ^3^Department of Computer Science, University of Bath, Bath, United Kingdom

**Keywords:** Twitter, emotion, lockdown, LIWC, circadian

## Abstract

Diurnal variations in indicators of emotion have been reliably observed in Twitter content, but confirmation of their circadian nature has not been possible due to the many confounding factors present in the data. We report on correlations between those indicators in Twitter content obtained from 9 cities of Italy and 54 cities in the United Kingdom, sampled hourly at the time of the 2020 national lockdowns. This experimental setting aims at minimizing synchronization effects related to television, eating habits, or other cultural factors. This correlation supports a circadian origin for these diurnal variations, although it does not exclude the possibility that similar zeitgebers exist in both countries including during lockdowns.

## 1 Introduction

It is known that the time series of Twitter content contains periodic patterns that are seen in the relative frequency of certain words (Golder and Macy, 2011). Special word lists commonly used to create psychometric indicators, for example for the basic emotions, are also known to produce periodic signals (e.g., LIWC, Linguistic Inquiry and Word Count, Pennebaker et al., 2015b). These indicators include anger, anxiety, sadness, negative emotion, and positive emotion, among others, and all contain a periodic component, along with a residual that is known to respond (also) to external events. For example, they show 24-h cycles, as well as responding in predictable manners to events such as disasters, weather, elections or sport events (Golder and Macy, 2011; Lansdall-Welfare et al., 2016; Dzogang et al., 2017a,b).

What has not been established is whether the periodic component is a reflection of external changes (as in the case of responses to food, weather or media content) or rather is mediated by internal factors, reflecting regulation of the affective state by the endogenous circadian clock which is in turn entrained by external zeitgebers. In other words, what is not clear is the mechanism by which the time series of individual words is synchronized, so that their simultaneous variation results in a periodic variation of the overall indicator [similar synchronization effects can also be seen in different types of data, e.g., in web searches or purchasing behavior (Dzogang et al., 2016, 2017b)].

A previous study (Wang et al., 2021) has shown that these diurnal variations have not been affected by the 2020 lockdown in the United Kingdom, lending some support to the conjecture that daily routines (such as work, commute, and school) are not directly responsible for changes in Twitter content, and may perhaps represent endogenous regulation from the central pacemaker in the brain.

However, that study by Wang et al. (2021) could still be influenced by a number of confounders. A confounder is a variable that influences both the dependent and independent variables, potentially leading to a spurious correlation. For instance, consider a scenario where a television show, watched by an entire nation and broadcast live, creates time-correlated popular reaction. Such traditional habits and routines, unaffected by the lockdown, could act as confounders. This may introduce noise to the study, as it may create correlations between time and emotion that are not directly related to the variables under investigation.

In the present study, we repeat the analysis of diurnal variation of psychometric indicators during the 2020 lockdown, but this time using Twitter content collected in Italy. Additionally, we used the same population as our previous study (Wang et al., 2021) in the United Kingdom for comparison. This approach is designed to remove more potential confounders and lend further support to the conjecture that these variations might be mediated by endogenous biological processes. Specifically, we aim to observe diurnal rhythms in expression of emotion in situations where daily routine is not affected by school, work and commute.

After the experiment, we observe that for most, but not all, of the emotion indicators, there is a significant diurnal component in both countries, and there is a significant correlation between the time series. Only in the case of one indicator this is not the case, and we will discuss possible explanations below. In the following sections, we will detail explain our research methodology, results and discussion about it.

## 2 Materials and methods

### 2.1 Ethics approval

Ethical approval for our Twitter research studies was provided by the Ethical Officer of the Faculty of Engineering of the University of Bristol. The data used is collected by periodically sampling anonymous tweets from a fixed set of broad locations, in order to extract word frequencies in aggregate, without keeping individual emails. No personal data is collected, no individual ID is kept, no inference is (or could be) made about individuals, the individual users are expected to change at every sample. We only release aggregated time series of word frequencies. This complies with the recommendations of Portaluppi et al. (2010).

### 2.2 Data collection

We collected Twitter content from 9 Italian cities every hour at the same time as the other collection in 54 United Kingdom cities.

At any given hour we analyse a “slice” of the population of active twitter users, rather than following any individuals, so that we have a cross-sectional sample. The users are not recorded and vary at each collection, a design choice used in several previous studies (Dzogang et al., 2017a,b) that enables us to access average patterns that appear across many individuals at the same time, while maintaining the data anonymous from the start, since words are only linked to locations and time intervals. We comply with Twitter’s Terms of Service.^1^ The information collected for each tweet is: anonymized textual content, location (within 10 km of one of the 9 urban centres in Italy and 54 urban centres in the United Kingdom), date and time of collection.

In Italy the 2020 national lockdown lasted from 9 March 2020 to 18 May 2020 (on this day the first shops reopened, but not the schools). In the United Kingdom the 2020 national lockdown lasted from 22 March 2020 to 30 May 2020. We have chosen to analyse data collected in the 10 weeks between March 22nd, 2020, and May 30th, 2020 (the duration of the United Kingdom lockdown, a time interval during which most schools and workplaces were closed in both countries).

We sample the 100 most recent tweets every 30 min from broad locations (cities or surrounding territory), without filtering for any specific hashtags or keywords, and then we aggregated the data to in order to obtain time intervals of 1 h.

The locations correspond to the largest 9 cities in Italy (*Bari, Florence, Bologna, Genoa, Palermo, Turin, Naples, Milan and Rome*) and 54 largest cities in the United Kingdom (*Aberdeen, Basildon, Belfast, Birmingham, Blackburn, Blackpool, Bolton, Bournemouth, Bradford, Brighton, Bristol, Cardiff, Coventry, Derby, Dundee, Edinburgh, Glasgow, Gloucester, Huddersfield, Hull, Ipswich, Leeds, Leicester, Liverpool, London, Luton, Manchester, Middlesbrough, Newcastle, Newport, Northampton, Norwich, Nottingham, Oldham, Oxford, Peterborough, Plymouth, Poole, Portsmouth, Preston, Reading, Rotherham, Sheffield, Slough, Southampton, Southend, Stockport, Stoke, Sunderland, Swansea, Swindon, Watford, Wolverhampton and York*).

Due to software legacy reasons, our data collection pipeline samples words by collecting 280-characters of each message in Italy, and 140 messages in the United Kingdom. However, from previous studies (Wang et al., 2021), we know that the 140-characters version gives equivalent results to the 280-characters version.

As part of our processing, we do not remove holiday greetings [something that was done in our previous studies where seasonality was investigated (Dzogang et al., 2017b)], and we use the content of retweets, after removing the token RT.

We report all results in local time. Because of the change to Daylight Saving Time in March, there is a one-hour gap on the time of March 29th, 2020, 02:00-03:00, so we used the average of this period (2 am–3 am) for the entire dataset to fill this gap and obtain 24 readings per each day.

This process produced 1,680 text samples for each country, obtained aggregating all tweets collected in that country within a given hour, over a 10-week period. These samples form two textual time series, the one from Italy combining a total of 2 M tweets and the one from the United Kingdom a total of 33 M tweets, collected during the same 70 days. The data is summarized in Table 1.

**Table 1:** Statistical summary of the two datasets.

Dataset name	United Kingdom data	Italian data
Sampling interval	March 22nd, 2020 – May 30th, 2020 (70 days)	
Sampling frequency	Hourly	Hourly
Number of time points	1,680	1,680
Size in words	510,640,042	61,278,060
Number of tweets	33,954,858	2,793,150
Locations	54 Largest urban centers in the United Kingdom	9 Largest urban centers in Italy
Tweet length	140 Characters	280 Characters

### 2.3 Data analysis

The textual time series described above are used to extract the hourly time series of six psychometric indicators (positive emotion (posemo), negative emotion (negemo), anger, anxiety, sadness and general affect.). Faced with the challenge of extracting LIWC indicators from Italian language time series, we had three potential options: using a list specifically handmade and validated for Italian language; translating an English language list into Italian; translating the Twitter content into English. As we could not find adequate Italian language lists, and the translation of individual words from the English is not recommended by the LIWC creators due to linguistic ambiguity (Boyd et al., 2022), we followed their advice and machine-translated the Twitter content from Italian to English, a process that has reached a high level of accuracy and can translate words in a way that is sensitive to the context in which they are used. While some “noise” is expected at each such processing step, we are only interested in detecting the presence of a periodic component in the resulting signal across many weeks.

The following methods describe how we processed and interpreted the data to identify key patterns and insights in our analysis.

#### 2.3.1 Extraction of psychometric indicators from English language text

##### 2.3.1.1 LIWC scores extraction

We extracted six psychometric indicators (positive emotion or posemo, negative emotion or negemo, anxiety, anger, sadness, affect) using the rules and the word lists described in LIWC2015 (Pennebaker et al., 2015a). In this way we obtained a numerical score for each emotion at each hour of the periods under investigation.

The text was preprocessed as follows: we lowered uppercase characters, tokenized text into words (alphanumeric strings, referred to as ‘tokens’), removed punctuation and emojis, etc.; each word was compared with the word lists and the rules lists of LIWC2015, incrementing the counter of each LIWC emotion indicator when a match occurred; then all those counts were normalized by using the total number of tokens (in that time interval), obtaining in this way a time series of relative frequency. In this step we ignored the 2-grams such as “kind of,” an approximation that in previous studies was found not to affect the signal extracted (in a sample of 10,000 documents we observed a correlation of 0.99 between the two methods).

##### 2.3.1.2 Example

The hypothetical hashtag #circadian_pattern would be tokenized into {circadian, pattern}, any emojis would be removed, the hypothetical URL http://www.circadian-pattern.com would be tokenized as follows: {“http,” “//www,” “circadian,” “pattern,” “com”}.

This process produced six LIWC scores for each hour of the 70 days interval, in other words a time series of length 1,680. As indicated by our data availability statement, these numerical time series, sufficient to reproduce the study, will be publicly available from the time of publication.

#### 2.3.2 Working with Italian tweets

In order to create a comparable time series from the collection of Italian tweets, we followed the recommendations of Boyd et al. (2022) and used a machine translation software to translate the Italian tweets into English, and then analysed the resulting dataset with the same LIWC word lists and software used for the English corpus.

For this purpose, we used Google Translate. While this cannot provide a perfect translation result of tweets, however we believe that this is the best of the available alternatives and is recommended by several authors (Windsor et al., 2019; Barbosa et al., 2021; Meier et al., 2021; Boyd et al., 2022). This is because the use of handmade Italian lists turned out not to be a viable option, after an inspection of the available such lists, which turned out to contain entirely different words from the English ones. At the same time, the automated translation of English lists into Italian would create a much higher level of noise than the translation of the Twitter corpus, since the translation of a word depends on the context, something that Google Translate can do when translating tweets but not when translating lists of words. Finally, the option of generating a brand-new list of Italian words for sentiment would have required a validation step on annotated Italian data, which would have been beyond the scope of this study.

#### 2.3.3 Time series analysis

We analyse the six-hourly time series in two ways: by computing their Fourier Spectrum and by creating an average 24 h (resp. 168 h) profile, following the same steps as in Wang et al. (2021), that is: we performed a Fast Fourier Transform (FFT) of the time series, and one of its random permutation (for statistical testing purposes, based on a null model of white noise), then we detected the peaks that were above “the noise level,” and had period lower than 7 days. Longer periods would be harder to estimate with the given dataset and would fall outside the scope of the present study.

##### 2.3.3.1 Spectral analysis

Following the recommendations of Portaluppi et al. (2010) we performed a Spectral Analysis of the Time Series by computing the FFT, which resulted in an estimate of the variance explained by each Fourier component. Peaks are found using the peak-finder algorithm in the *scipy* library of Python.

We consider significant those peaks whose energy is higher than the maximum observed in randomized data, and for those we report the frequency and the fraction of variance explained (Achen, 1990). Then we modelled our time series as a repetition of a 24 h (resp. 168 h) waveform, by computing an average 24 h (resp. 168 h) profile, which we call average daily profile (resp. Average Weekly Profile) and indicate with ADP (resp AWP). The addition of a Weekly Profile is to visualize possible periodic effects on the weekends.

In the computation of the average profiles, we estimated the confidence in our estimation of the mean by using the standard error of the mean (SEM) confidence intervals represent two standard errors of the mean (SEM). We quantify the extent to which a time series can be modelled as a repetition of identical 24 h cycles, by measuring the correlation between the original series and one generated by such a model (note: the square of this quantity also relates to the fraction of variance explained by our model). Value of *p*s for Pearson correlation coefficient are calculated with Python, using standard *t*-test.

##### 2.3.3.2 Average profiles for analysis of variance

We are interested in estimating how much of the overall variance can be explained by a model where the time series is approximated as the repetition of 24 h or 7 days “profiles,” which we call average daily profile (ADP) and average weekly profile (AWP).

We generate a periodic time series, by concatenating average daily profiles, to have a baseline time series, and measure the variance explained by it. This is done by repeating the same 24 h pattern (average daily profile, or ADP, obtained by computing the average value for each hour along the entire time series). We do the same with a 7 days (168 h) model, in which we compare average weekly profiles (AWP), to account for the possibility that different weekdays might have different behavior. This is because the Fourier Spectrum of LIWC indicators is not monochromatic, despite having a strong 24 h component.

These profiles are defined by averaging every day from midnight to 11 pm (respectively, every week, from Sunday midnight to Saturday 11 pm). Note that, due to the possible difference between days, the AWP is not just the concatenation of 7 ADPs.

The ADP and AWP allow us to compute – for example-the 24 h average profile for a given indicator in Italy or in the United Kingdom, with their confidence bars, and to quantify the extent to which the time series can be described by a periodic model: each original sequence can be compared with the sequence that would be obtained by repeating the 24 h (resp.168 h) profile.

## 3 Results

### 3.1 Sanity check

#### 3.1.1 Sanity check – breakfast

**Figure 1:**
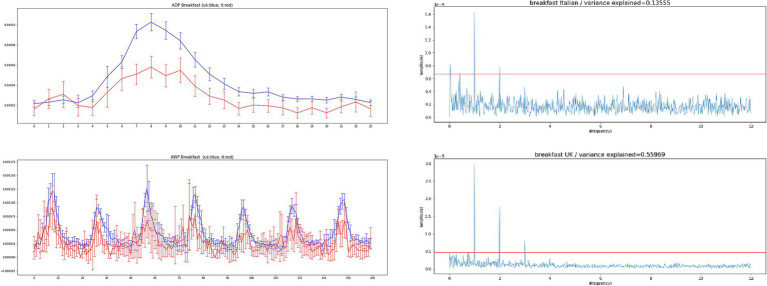
Top-left – the average daily profiles (ADP) for the word breakfast in the United Kingdom (blue), based on 140 Chars database and the word colazione in Italy (red), based on 280 Chars database for period (March 22, 2020 – May 30, 2020). Bottom-left – the average weekly profiles (AWP) for the word breakfast in the United Kingdom (blue), based on 140 Chars database and the word colazione in Italy (red), based on 280 Chars database for period (March 22, 2020 – May 30, 2020). Confidence intervals represent two standard errors of the mean (SEM). The correlation between the whole time series is 0.31, with a value of *p* lower than 0.001. The ADP profiles have correlation over 0.9. Right – the FFT spectrum for the Italian and English time series of the word breakfast, the red line marks the level of peaks obtained from randomly permuted data. Strong 24 h components can be seen in both time series, as well as 12 h components. An infradian peak is seen in Italian, but not in English, and is probably due to smaller data sizes in the Italian data.

We perform a sanity check of our software by comparing the 24 h profile and 168 h profile of select words that we expected to be the same between both countries, in order to provide context to interpret the results for the emotion indicators. A good example is the word breakfast which we expected to exhibit the same shape between the United Kingdom and Italy. This is a word that has virtually no translation ambiguity. Figure 1 shows that the word breakfast have same peak in the morning in the United Kingdom and in Italy, both in ADP and AWP, and similar Fourier spectrum.

### 3.2 LIWC psychometric indicators

#### 3.2.1 Fourier analysis

**Figure 2:**
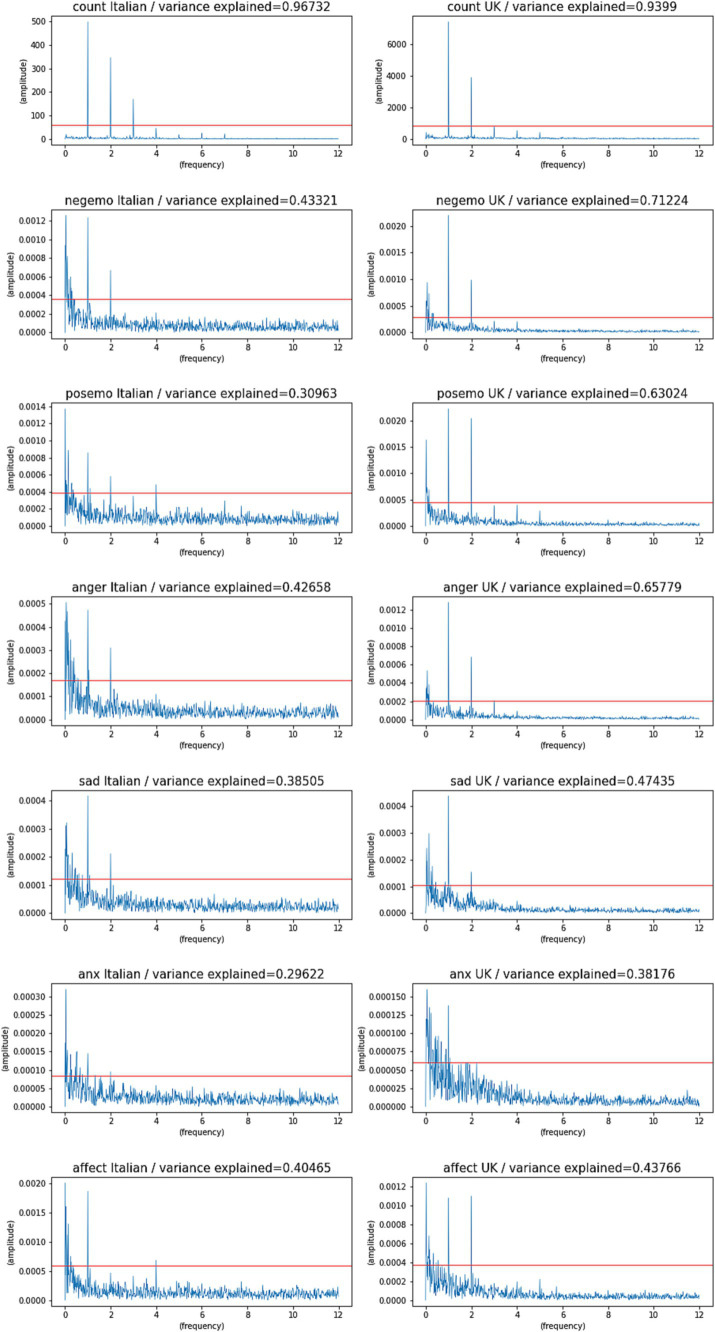
FFT spectra for all indicators and both countries [Italy (Italian) and United Kingdom (English)], in 2020 with 280 char Tweets. The red line indicates the largest value obtained by randomizing that time series (white noise model). The FFT spectra show frequencies in Hz on the x-axis, and their amplitudes on the y-axis. Frequencies below 1 Hz represent periods over 24 h (infradian). Frequencies above 1 Hz represent periods below 24 h (ultradian).

The FFT spectra for both datasets (Figure 2) show that for all the indicators there is a 24-h component, although with very different energy. For comparison we added the spectrum for the indicator “Counts,” that is for the time series of the number of tweets collected per time interval, which is very close to an idealized periodic behavior. The indicators based on word frequencies, on the other hand, suffer from various sources of noise, but still display significant periodic structures.

For simplicity, Table 2 reports the fraction of variance explained by the three main components of the Fourier Spectrum of each time series (that are above the significance threshold and have a period lower than 7 days). The FFT spectra in Figure 2 report frequencies in Hz on the x axis, and their amplitudes on the y axis. Frequencies below 1 Hz represent periods over 24 h (infradian). Frequencies above 1 Hz represent periods below 24 h (ultradian). The table reports these components as the Period of the oscillation (1 corresponding to 24 h, 0.5 corresponding to 12 h, etc.). As in previous studies (Wang et al., 2021), we only consider periods of 7 days or less, as longer periods are harder to estimate with a short time series and are more likely to reflect noise or longer trends in the data that are not of interest to this study.

**Table 2:** The three largest Fourier components and the variance explained by them (omitting components below the threshold and periods over 7 days) – for the dataset of 280 char Tweets collected in Italy and 140 char Tweets collected in United Kingdom.

LIWC indicator	Italian		United Kingdom	
	Component (TOP3)	Variance explained	Component (TOP3)	Variance explained
Count	1.0000	0.5987	1.0000	0.7363
	0.5000	0.2695	0.5000	0.2036
	0.3333	0.0766	0.3333	0.0083
Negemo	4.1176	0.0238	7.0000	0.0523
	0.5000	0.0297	1.0000	0.4604
	1.0000	0.1010	0.5000	0.1056
Posemo	7.0000	0.0476	7.0000	0.0185
	1.0000	0.0444	1.0000	0.3022
	0.5000	0.0204	0.5000	0.2761
Anger	7.0000	0.0362	7.0000	0.0319
	4.1176	0.0304	1.0000	0.4223
	1.0000	0.0570	0.5000	0.1287
Sadness	3.1818	0.0241	7.0000	0.0766
	1.0000	0.0914	1.0000	0.1866
	0.5000	0.0234	0.5000	0.0270
Anxiety	3.8889	0.0205	6.3636	0.0234
	1.9444	0.0227	4.3750	0.0263
	1.0000	0.0213	1.0000	0.0224
Affect	7.0000	0.0464	7.0000	0.0357
	4.1176	0.0154	1.0000	0.1761
	1.0000	0.0946	0.5000	0.1723

The infradian peaks can be explained with noise and small data sizes, as discussed in the text.

Table 2 reports the period and variance for the main peaks in FFT of each time series (it only reports the three largest peaks that are also larger than the random baseline, while having periods lower than 7 days). Figure 2 shows the complete spectrum of each time series. Despite our statistical filtering procedures, we do see some periods of 4 or 5 days in the Italian data, which are the likely result of higher noise levels and much smaller sample sizes in this dataset. This was further supported by our tests, which reproduced the same effect on United Kingdom data by simply reducing the sample sizes, and we therefore will not consider them of biological significance.

In the case of Italian data, there is higher noise, due both to the much smaller sample sizes involved, and to the ambiguity inherent in the machine translation process. This makes the Italian spectra for certain indicators much noisier and more difficult to filter or explain. However, the diurnal (24 h) component is the largest one for Count (60% of variance explained), Negemo (10%), Sadness (9%), Affect (9%). But it is only the second largest component for Posemo (4%); also, it is the third largest component for Anger (6%), it is very weak for Anxiety (explaining only 2% of its variation) which is the noisiest indicator in both languages. Some of these less periodic indicators show components different from the 12 h, 24 h and 7-days periods that are seen in most of the United Kingdom data. Anxiety in particular does not seem to follow a clear circadian pattern.

The English data, instead showed higher scores, indicating lower noise levels. The 24-h component accounts for 74% of the variance in “Count,” followed by Negemo (46%), Sadness (19%), Affect (17.6%); Posemo (30%), Anger (42%), Anxiety (2.2%; see Table 2 for details about the other spectral components).

Both ultradian (12 h) and infradian (7 days) components are sometimes present: ultradian effects could be reflecting the shape of the waveform, rather than actual biological events (since the series is not monochromatic). Infradian effects with a length over a week are difficult to estimate with confidence in a 10-week long time series, and may reflect overall trends in the time series, and are removed in this analysis. As discussed above, the presence of some infradian components is the likely a result of noise and small sample sizes.

Some of the indicators also have a significant 12 h component, which – in the case of Italian - explains 27% of variation for Count, 3% for Negemo, 2% for Posemo and 2% for Sadness. It is not above our significance threshold for Anger, Anxiety and Affect. We also see a 7-day component in Posemo (5%), Anger (3%), Affect (4%). In the English language data, the 12 h component is observed in negemo, posemo, anger, affect.

#### 3.2.2 AWP-ADP analysis of variance

**Figure 3:**
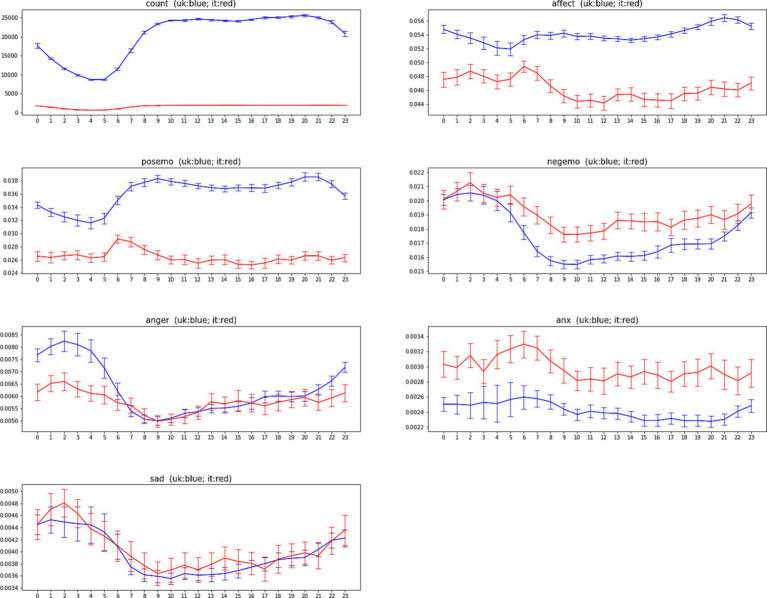
The average daily profiles (ADP) of LIWC2015 indicators for Italy (red) and United Kingdom (blue) for the period from March 22, 2020, to May 30, 2020. Confidence intervals represent two standard errors of the mean (SEM).

**Figure 4:**
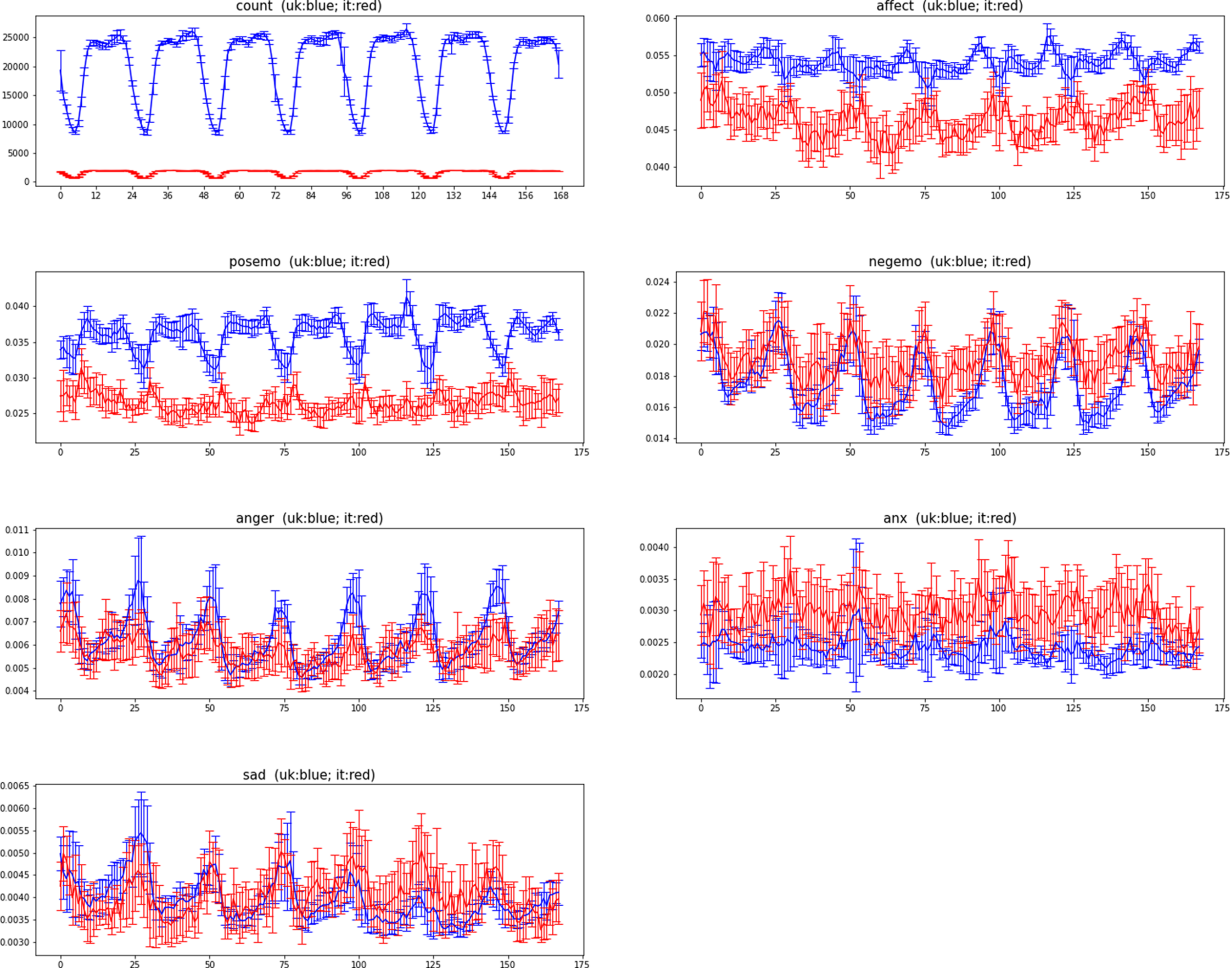
The average weekly profiles (AWP) of LIWC2015 indicators for Italy (red) and United Kingdom (blue) for period (March 22, 2020 – May 30, 2020). Confidence intervals represent two standard errors of the mean (SEM).

We report in Figures 3, 4 the ADP and AWP (along with their confidence bars) used to compare the baseline temporal variations of LIWC indicators in Italy and in the United Kingdom.

Figure 3 shows the ADP and its confidence bars for each of the indicators, for each of the two countries. Figure 4 shows the same for AWP. In order to quantify how similar the Italian data and the United Kingdom’s data are, we have measured the correlation between Italy’s time series and United Kingdom’s time series. Table 3 shows the correlation between the United Kingdom’s data and Italian’s data.

**Table 3:** The Pearson Correlation Coefficient (and value of *p*) between Italian data and United Kingdom’s data, based on 280 Chars database (140 Chars database for United Kingdom’s), for the period from March 22, 2020, to May 30, 2020.

Correlation coefficient between Italy (translated to English) and the United Kingdom		
	Correlation	Value of *p*
Count	**0.9354**	<0.001
Affect	0.0407	0.096
Posemo	**0.0803**	<0.001
Negemo	**0.2644**	<0.001
Anger	**0.2603**	<0.001
Anxiety	**0.1320**	<0.001
Sadness	**0.1233**	<0.001

Value of *p*s for the Pearson correlation coefficient are calculated automatically with Python, using the standard *t*-test. The bold value in these tables means the value is significantly correlated.

#### 3.2.3 Daily models

To quantify the extent to which a time series can be modeled as a repetition of identical 24 h cycles, we have measured the correlation between the original series and one generated by such a model. We perform this for: ADP Italy and United Kingdom (Table 4). Value of *p*s for Pearson correlation coefficient are calculated automatically with Python, using standard t-test.

**Table 4:** The Pearson correlation coefficient (and value of *p*) between the original data and the data obtained from the periodic model, based on 280 Chars database (140 Chars database for the United Kingdom’s), for the period from March 22, 2020, to May 30, 2020.

24 h periodicity 2020	Italy (Italian)		United Kingdom (English)	
	Correlation	P-Value	Correlation	P-Value
Count	**0.9870**	<0.001	**0.9767**	<0.001
Affect	**0.3507**	<0.001	**0.4325**	<0.001
Posemo	**0.3178**	<0.001	**0.6689**	<0.001
Negemo	**0.3720**	<0.001	**0.7065**	<0.001
Anger	**0.2947**	<0.001	**0.6821**	<0.001
Anxiety	**0.2041**	<0.001	**0.2061**	<0.001
Sadness	**0.3449**	<0.001	**0.4367**	<0.001

*P*-values for the Pearson correlation coefficient are calculated automatically with Matlab, using the standard *t*-test. The bold value in these tables means the value is significantly correlated.

We observe that the time series remains the same pattern between Italy and the United Kingdom. Except for Positive Emotion, all other LIWC indicators have the same shape in these two countries. Most of the indicators have a strong and significant correlation with their 24-h model, the indicator Anxiety has the lowest level of correlation with a daily model.

#### 3.2.4 Weekly models

We have measured the correlation between the original series and one generated by such a model, in order to quantify the extent to which a time series can be explained as a repetition of identical 168 h cycles. We perform this for: AWP Italy and United Kingdom (Table 5).

**Table 5:** The Pearson correlation coefficient (and value of *p*) between original data and model periodic data, based on 280 Chars database (140 Chars database for the United Kingdom’s), for period (March 22, 2020 – May 30, 2020).

168 h periodicity 2020	Italy (Italian)		United Kingdom (English)	
	Correlation	*p*-value	Correlation	*p*-value
Count	**0.9895**	<0.001	**0.9811**	<0.001
Affect	**0.4606**	<0.001	**0.5056**	<0.001
Posemo	**0.4648**	<0.001	**0.7026**	<0.001
Negemo	**0.4316**	<0.001	**0.7562**	<0.001
Anger	**0.4034**	<0.001	**0.7148**	<0.001
Anxiety	**0.3290**	<0.001	**0.3038**	<0.001
Sadness	**0.4292**	<0.001	**0.5982**	<0.001

*P*-values for Pearson correlation coefficient are calculated automatically with Matlab, using standard *t*-test. The bold value in these tables means the value is significantly correlated.

Most indicators have remained periodic also between Italy and the United Kingdom during the lockdown period to different extents (except for the indicators posemo and affect). As for ADP models, also for AWP models anxiety is the indicator which correlates the least with a weekly model, while the remaining indicators show a larger effect size and strong statistical significance.

## 4 Discussion

Our finding of similar diurnal patterns of emotional indicators in two culturally and linguistically different countries lends more support to the proposal that there is a common origin for these oscillations most likely reflecting the circadian output of the hypothalamic suprachiasmatic nucleus-the body clock, which is of course itself synchronized by sunlight.

Despite the approximations introduced by the translation process, and by the use of simple word frequencies as proxies for sentiment, all signals are found to be periodic in both United Kingdom and Italy, and most of them show a significant correlation across the two countries. As a sanity check, we observe that the time series of Twitter volume has a correlation coefficient of 0.93, meaning that people in the two countries tended to tweet with the same frequency across the day. Furthermore, using the word “breakfast” as an additional “sanity check,” we see that the smaller data sizes in the Italian collection do affect the quality of the analysis, resulting in higher noise levels, even for a word with virtually no translation ambiguity such as “colazione/breakfast.” Higher noise can be expected from the longer lists used by LIWC, which were not selected with translation ambiguity in mind. Despite the obvious limitations of this procedure, clear periodic components can be observed in Twitter content collected in Italy, for most indicators of sentiment.

Regular diurnal variations in Twitter were reported over 10 years ago by Golder and Macy (2011) and confirmed by our previous studies (Dzogang et al., 2017a,b, 2018; Wang et al., 2021). In most indicators, there is a significant correlation between time series computed from the Italian and United Kingdom data, suggesting that the diurnal changes in expression of emotion happen at the same time of day in both countries. Furthermore, many possible “social” sources of synchronization were removed by the lockdowns of spring 2020, without affecting the general periodic structure of those time series (Wang et al., 2021). In this study we show that these patterns are also stable across countries and languages, even during the lockdown period.

For all the negative emotions there appears to be a powerful time dependent driver for their diurnal variation, and that this factor can be observed not only during normal times but also during lockdown and also in both countries. This applies to Anger (0.26), Anxiety (0.13) and sadness (0.12). Although we cannot discount the effects of external influences such as food, television, exercise and light, the commonality of these rhythms across countries and between normal times and lockdown suggests a more intrinsic circadian regulation of negative affect.

Positive emotions (posemo) show a very low correlation coefficient of 0.08 (which is still significant according to the t-test), whereas affect is not significantly correlated. It is unclear why we should have this discrepancy. One possibility is that the two countries simply did not have a correlated expression of positive emotion, which would be an interesting finding, since this might reveal that this method is predominantly influenced by non-circadian factors. The other is that enough words in the posemo list were not robust to machine-translation approximation, resulting in too noisy a signal to give a significant correlation. We have also observed that using lower data sizes results in increased noise in the FFT spectrum, which explains the presence of some infradian peaks in the Italian time series.

An interesting and complementary study was recently published (Metzler et al., 2023), comparing the average LIWC scores in Twitter content during the pandemic and before it, in 6 language and 18 countries. This study did not focus on circadian variations in emotion, but rather in comparing average levels, to establish the effect of the pandemic on collective mood. They found that anxiety and sadness had increased on average, while anger had decreased. These results are compatible with those of our earlier paper (Wang et al., 2021) which compared these quantities for the United Kingdom only, finding that indeed anger had decreased, sadness had increased, while in our case the changes in anxiety were within the error bars.

In conclusion, we have found that regular diurnal variations in emotional indicators in Twitter are similar across cities in the United Kingdom and Italy-both during normal times and during lockdown. Although we cannot exclude common external influences in all these situations, these data do add to the evidence for a circadian regulation of emotional expression.

## Data availability statement

The datasets presented in this study can be found in online repositories. The names of the repository/repositories and accession number(s) can be found below: https://osf.io/jqey6/.

## Author contributions

SW: Data curation, Formal analysis, Software, Visualization, Writing – original draft. SL: Conceptualization, Methodology, Writing – original draft, Writing – review & editing. NC: Conceptualization, Formal analysis, Methodology, Supervision, Writing – original draft.

## Footnotes

^1^https://twitter.com/en/tos

## References

[ref1] Achen C. H. (1990). What does "explained variance" explain? – reply. Polit. Anal. 2, 173–184. doi: 10.1093/pan/2.1.173

[ref4] Barbosa A. Ferreira M. Ferreira Mello R. Dueire Lins R. Gasevic D. (2021). The Impact of Automatic Text Translation on Classification of Online Discussions for Social and Cognitive Presences. LAK21: 11th International Learning Analytics and Knowledge Conference, 77–87.

[ref5] Boyd R. L. Ashokkumar A. Seraj S. Pennebaker J. W. (2022). The Development and Psychometric Properties of LIWC-22. Austin, TX: University of Texas at Austin.

[ref7] Dzogang F. Goulding J. Lightman S. Cristianini N. (2017b). “Seasonal variation in collective mood via Twitter content and medical purchases” in Advances in intelligent data analysis XVI. IDA 2017. Lecture Notes in Computer Science. eds. Adams N. Tucker A. Weston D., vol. 10584 (Cham: Springer)

[ref8] Dzogang F. Lansdall-Welfare T. Cristianini N. (2016). "Seasonal Fluctuations in Collective Mood Revealed by Wikipedia Searches and Twitter Posts," 2016 IEEE 16th International Conference on Data Mining Workshops (ICDMW), Barcelona, pp. 931–937.

[ref9] Dzogang F. Lightman S. Cristianini N. (2017a). Circadian mood variations in Twitter content. Brain Neurosci. Adv. 1:2398212817744501. doi: 10.1177/2398212817744501 29270466 PMC5736128

[ref10] Dzogang F. Lightman S. Cristianini N. (2018). Diurnal variations of psychometric indicators in Twitter content. PLoS One 13:e0197002. doi: 10.1371/journal.pone.0197002, PMID: 29924814 PMC6010242

[ref11] Golder S. A. Macy M. W. (2011). Diurnal and seasonal mood vary with work, sleep, and daylength across diverse cultures. Science 333, 1878–1881. doi: 10.1126/science.1202775, PMID: 21960633

[ref14] Lansdall-Welfare T. Dzogang F. Cristianini N. (2016). Change-Point Analysis of the Public Mood in UK Twitter during the Brexit Referendum. In 2016 IEEE 16th International Conference on Data Mining Workshops (ICDMW). p. 434–439.

[ref15] Meier T. Boyd R. L. Mehl M. R. Milek A. Pennebaker J. W. Martin M.. (2021). (Not) lost in translation: psychological adaptation occurs during speech translation. Soc. Psychol. Personal. Sci. 12, 131–142. doi: 10.1177/1948550619899258

[ref16] Metzler H. Rimé B. Pellert M. Niederkrotenthaler T. Di Natale A. Garcia D. (2023). Collective emotions during the COVID-19 outbreak. Emotion 23, 844–858. doi: 10.1037/emo0001111 35787108

[ref18] Pennebaker J. W. Boyd R. L. Jordan K. Blackburn K. (2015a). The Development and Psychometric Properties of LIWC2015. Austin, TX: University of Texas at Austin

[ref19] Pennebaker J. W. Booth R. J. Boyd R. L. Francis M. E. (2015b). Linguistic Inquiry and Word Count: LIWC 2015. Austin, TX: Pennebaker Conglomerates.

[ref20] Portaluppi F. Smolensky M. H. Touitou Y. (2010). Ethics and methods for biological rhythm research on animals and human beings. Chronobiol. Int. 27, 1911–1929. doi: 10.3109/07420528.2010.516381, PMID: 20969531

[ref25] Wang S. Lightman S. Cristianini N. (2021). Effect of the lockdown on diurnal patterns of emotion expression in Twitter. Chronobiol. Int. 38, 1591–1610. doi: 10.1080/07420528.2021.1937198, PMID: 34134583

[ref26] Windsor L. C. Cupit J. G. Windsor A. J. (2019). Automated content analysis across six languages. PLoS One 14:e0224425. doi: 10.1371/journal.pone.0224425, PMID: 31747404 PMC6867602

